# Longing for touch post-COVID-19: current observations and future directions

**DOI:** 10.1038/s41598-023-49113-1

**Published:** 2023-12-13

**Authors:** Birgit Hasenack, Larissa L. Meijer, Anna van Harmelen, Krista E. Overvliet, Anouk Keizer

**Affiliations:** 1 Faculty of Social and Behavioural Sciences, Experimental Psychology, Utrecht University, Utrecht, The Netherlands; 2 Faculty of Social and Behavioural Sciences, Clinical Psychology, Utrecht University, Utrecht, The Netherlands

**Keywords:** Psychology, Human behaviour

## Abstract

Previous studies have reported an association between the COVID-19 pandemic, social distancing regulations and longing for touch (LFT; i.e., a discrepancy between actual touch frequency and one’s desire to be touched). However, less is known about the prevalence and severity of LFT in the general population in the absence of social distancing regulations. The aim of this study was therefore to exploratively compare data collected during and after the pandemic. Pandemic data was collected online in an international sample (n = 1982), of which a matched subsample (n = 115) was used in the reported analyses. Post-pandemic data was collected one week after social distancing regulations restrictions were lifted in the Netherlands (n = 60) and when virtually no restrictions were in place (n = 55). The severity of LFT was significantly higher during the pandemic than afterwards. Although there were no significant differences in the general prevalence of LFT, significantly more participants reported high levels of LFT (score of 75–100) during the pandemic. We cautiously conclude that, although LFT may have peaked during the pandemic, a large portion of the general population desires to experience more interpersonal touch, even in the absence of social distancing regulations.

## Introduction

To limit the spread of the COVID-19 virus various restrictions were imposed world-wide^1^ between 2020 and 2023. These restrictions significantly limited opportunities for interpersonal physical contact. Recent studies show that, as a result of the regulations, a large portion of the general population experienced longing for touch (LFT^2–4^).

LFT, or touch deprivation, refers to an imbalance between the amount of touch an individual wishes to receive and their actual touch frequency^5^. Previous research has shown that LFT is associated with a variety of negative outcomes, including increased levels of stress, lower psychological resilience and difficulties in coping with stress^6^. In addition, LFT was found to be associated with lower levels of physical, social and psychological quality of life during the pandemic^2^. Given the potential consequences of LFT, it is important to investigate whether feelings of touch deprivation remain prevalent in the absence of COVID-19 regulations. Although this is understudied, there are indications that LFT is indeed not a problem exclusively associated with the pandemic. A pre-pandemic study by Beßler et al.^5^ reported that 72.7% of a German community sample experienced varying degrees of LFT. More research is clearly warranted since this is the only study to report LFT prevalence outside of the pandemic, and there are no studies that have reported post-COVID data.

The aim of this brief report is therefore to explore the severity and prevalence of LFT post-COVID-19. By doing this, we aim to observe to what extent the general population continues to expereince LFT. In the absence of longitudinal studies, post-COVID data will also be exploratively compared to data collected during the pandemic. Although options to compare data from different samples are limited, this comparison was included to put the post-COVID-19 data in perspective. The pandemic data used in this study was collected from April to October 2020, when strict restrictions were in place (for a full description, see Meijer et al.^3^). Post-pandemic data was collected in the Netherlands at two different timepoints: one week after social distancing regulations were lifted, between September 30 and October 3 2020 and when virtually no restrictions were in place, between October 2022 and February 2023. Pandemic and post-pandemic data will be exploratively compared with respect to both the severity and prevalence of LFT.

## Materials and methods

### Participants

The sample characteristics for each study are included in Table 1. The sample of the study by Meijer et al.^3^, hereafter referred to as the *During-COVID* study, consisted of 1982 participants (age *M* = 38.53, *SD* = 15.62, 79.7% identified as female). The majority of the participants resided in the Netherlands at the time of the study (68.1%). Participants were included in the main analysis if they were older than 16, and if they reported not having been diagnosed with a psychiatric or neurological disorder.

**Table 1:** Sample characteristics of the included studies.

	N	Age (SD)	Age (min–max)	Gender (% female)
Meijer et al. (2022) (during-COVID)				
Original sample	1982	38.53 (15.62)	16–87	79.7
Subsample*	115	31.33 (12.25)	17–74	81.7
Post-COVID1	60	36.32 (12.79)	17–74	65
Post-COVID2	55	25.89 (8.94)	18–57	100

*A matched subsample was selected from the original dataset for data-analysis. A detailed explanation of this procedure can be found under Data-analysis.

The sample of the first post-COVID study (*Post-COVID1*), consisted of 60 participants (age *M* = 36.32; *SD* = 12.79, 65% identified as female). All participants were above 16 years of age and were recruited during two different science festivals in the Netherlands (Betweter Festival and Weekend of Science). Participants with a self-reported diagnosis of a psychiatric or neurological disorder were excluded from the primary analyses.

The sample of the second post-COVID study (*Post-COVID2*) consisted of 55 female participants (age *M* = 25.89, *SD* = 8.94), who were all above the age of 18. Participants were recruited at Utrecht University and within the social network of the researchers. Similar to the aforementioned studies, a self-reported history or current psychiatric or neurological illness was used as exclusion criteria.

All three studies were approved by the local faculty ethical review board of Utrecht University (protocol numbers 20-210, 21-0317 and 22-0425, respectively), and executed in accordance with the corresponding guidelines and regulations.

### Longing for Touch Questionnaire

The Longing for Touch Questionnaire (LFTQ) contains two items (“*Currently I would prefer to be touched by others …*” and “*Currently I would prefer to touch others …*”). Participants use a Visual Analogue Scale (VAS) to answer these questions, with responses ranging from 0 (“*Currently I would prefer to be touched less by others/to touch others less*”) to 100 (“*Currently I would prefer to be touched more by others/to touch others more*”). An average LFT score was subsequently calculated by taking the mean across the two items, with scores above 50 being indicative of a longing for touch. This cut-off is based on the definition of LFT, which includes an imbalance between the desire to be touched and actual touch frequency. This means that people with LFT are expected to indicate a desire to be touched more (i.e., scores above 50) rather than less (i.e., scores below 50). An average score of 50 suggests satisfaction with the amount of touch that is received by the participant. The reliability of this questionnaire was high in previous studies^2,3^ and in the current sample, Cronbach’s α = 0.92.

The LFTQ used in the post-pandemic studies was an adapted version of the questionnaire used in the During-COVID study^2,3^. The primary difference was the range: the initial version of the LFTQ used a continuous scale from 0 to 10^2,3^, whereas the current version ranged from 0 to 100. In both cases, responses were rounded to integer values.

### Procedure

Data for the *During-COVID* study was collected online via Qualtrics between April 5th and October 8th 2020. All participants provided written consent at the start of the study. They subsequently provided demographic information, including gender and age, and filled out the LFTQ. This study included additional measurements that are not relevant to the aim of the current study. As such, they are not reported here, but an extensive overview can be found in previous publications^2,3^. Participants did not receive any form of compensation for their participation.

Data for the *Post-COVID1* study was collected at the Betweter Festival and at the Weekend of Science in the Netherlands between September 30, 2021 and October 3, 2021. These festivals both took place one week after the release of almost all COVID-19 related restrictions in the Netherlands. During this time face masks were only required in public transport, and a digital ‘COVID19 certificate’ was required to visit large events. At the start of the experiment, participants provided written informed consent and filled out demographic information about their gender and age. Afterwards, they completed the LFTQ. Participants did not receive compensation for their participation.

Data for the *Post-COVID2* was collected online via Qualtrics. At the time of the study (October 2022–February 2023), all COVID-19 related restrictions had been lifted in the Netherlands. After providing informed consent, participants answered questions about their age and gender. They subsequently filled out the LFTQ. Other measures were included in this study, but as these are not relevant to the aim of the current study, they are not reported here. Participants were mostly recruited from a student sample at the University of Utrecht, and students received credits in return for participating.

### Data analysis

Data about the severity of LFT was analysed using R in the Rstudio environment, using the ‘openxls’ package for reading MS Excelfiles, ‘tidyverse’ to sort, summarize and plot the data, ‘MatchIt’ to resample the data, and ‘stats’ and ‘afex’ to run statistical tests. The number of participants in the *During-COVID* sample was a multitude of the *Post-COVID1* and *Post-COVID2* samples. We therefore took a subsample of the *During-COVID* participants that matched both *Post-COVID* participant samples. To do so, we performed genetic matching with covariates age and gender, using the ‘MatchIt’ package^7^, which in turn calls functions from the ‘Matching’ package^8,9^. The data of 115 matched participants of the During-COVID (mean age: 31.33, range 17–74), 94 female, 21 male) and all the participants of both *Post-COVID* samples were used for further analysis.

We first calculated an LFT score by averaging the scores of the two items of the LFTQ. Participants with an average score of above 50 were classified as having LFT, as this signals an average desire to be touched and to receive touch more. Those with scores of 50 or below were classified as not having LFT. This reflects contentment with the actual touch- frequency, or a wish to be touched less, respectively. Furthermore, an average LFT score higher than 50 and lower or equal to 75 was categorized as low LFT and an average score of higher than 75 as high LFT. *During-COVID* data was collected on a continuous 1–10 scale^3^, whereas a 1–100 integer scale was used in the post-pandemic studies. As such, all scores were first normalized to a 1–100 scale^10,11^. In the *During-COVID* study a proration method was used to deal with missing data on the LFTQ. This decision was based on the high internal consistency reliability and item-total correlation of the LFTQ^12^. Proration was preferred over deletion to prevent unnecessary loss of power and data. The same method was therefore used in the analysis of *Post-COVID1* data, where 6 participants had missing values on one of the LFTQ items. There were no missing values in the *Post-COVID2* data.

A Shapiro–Wilk test showed that the data was not normally distributed (W = 0.95, *p* < 0.001), we therefore used a generalized linear model with Chi-Square test to check for a main effect of timepoint (*During-COVID*, *Post-COVID1*, *Post-COVID2*) and potential additional covariate effects for age and gender. Please note that the *Pre-COVID* study^5^ was not included in data analysis as that study used a different questionnaire to assess LFT severity.

Chi-square tests were used to compare the prevalence of LFT across the three studies. Separate analyses were conducted for the prevalence of general (scores > 50), low (scores 50–75) and high (scores 75–100) LFT.

## Results

**Figure 1:**
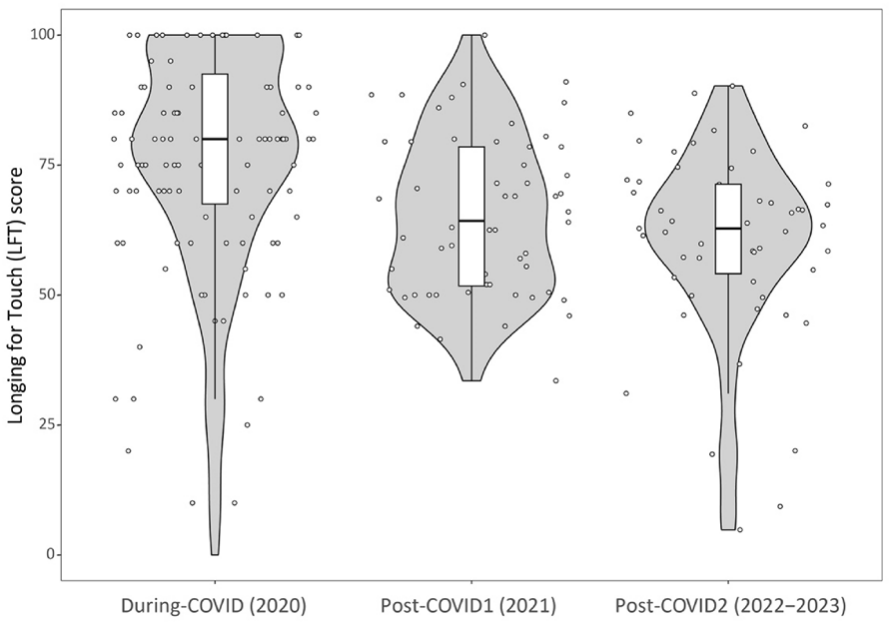
Boxplots representing the longing for touch (LFT) score for each time point, combined with violin plots, representing the distribution of the data and jittered points representing individual participants. The data in the “During-COVID (2020)” boxplot is based on the matched sample.

The mean LFT score for each of the studies is shown in Fig. 1. There was a main effect for timepoint on LFT score, but no effects for covariates age and gender were found(Table 2). Pairwise comparisons showed that the LFT severity *During-COVID* (*M* = 75.9 *SD* = 22.1) was significantly higher compared to both *Post-COVID1* (*M* = 65.21, *SD* = 15.04, *p* < 0.001) and *Post-COVID2* (*M* = 60.25, *SD* = 18.07, *p* < 0.001) (Fig. 1, Table 2). However, *Post-COVID1* and *Post-COVID2* were not significantly different from each other (*p* = 1).

**Table 2:** Results of the X^2^ test with factor timepoint and covariates age and gender.

	Df	Deviance	Residual df	Residual deviance	*p*
Timepoint	2	10,538.0	227	86,656	< 0.001
Age	1	246.5	226	86,409	0.423
Gender	1	60.7	225	86,349	0.691

The LFT prevalence (% participants with a LFT score higher then 50) was 80% for *Post-COVID1* and 78.2% for *Post-COVID*2, compared to the 86.1% observed in the current matched sample of the *During-COVID* study (Table 3)^5^. Following the distribution of the datapoints in the violin plot, a distinction was made between participants who experienced higher (75–100) and lower (50–75) levels of LFT. The prevalence of lower levels of LFT differed significantly between the datapoints (*p* < 0.001). Pairwise comparisons showed that during COVID (29.6%) there were significantly less participants with a lower LFT, compared to *Post-COVID1* (53.3%, *p* < 0.01) and *Post-COVID2* (61.8%, *p* < 0.001). There was no significant difference between *Post-COVID1* and *Post-COVID2* *(p* > 0.05). There was also a significant difference in the prevalence of higher LFT (*p* < 0.001). The prevalence was higher during COVID (56.5%) than in *Post-COVID1* (26.7%, *p* < 0.001) and *Post-COVID2* (16.4%, *p* < 0.001). There was no significant difference between *Post-COVID1* and *Post-COVID2* (*p* > 0.05). When looking at the overall LFT scores without making the distinction (> 50), no difference was found between the pandemic and post-pandemic studies (*p* > 0.05).

**Table 3:** LFT prevalence and severity.

	LFT prevalence (scores > 50) (%)	Low LFT prevalence (scores 50–75) (%)	High LFT prevalence (scores 75–100) (%)	LFT severity (SD)
During-COVID (matched subsample)	86.1	29.6	56.5	75.9 (22.1)
Post-COVID1	80	53.3	26.7	65.21 (15.04)
Post-COVID2	78.2	61.8	16.4	60.25 (18.07)

## Discussion

The aim of the current study was to shed light on post-pandemic LFT, and to exploratively compare this to data collected during the COVID-19 pandemic. We observed that LFT severity was significantly lower in post-pandemic than during the pandemic. There was no significant difference in LFT severity scores between the two post-pandemic studies. This suggests that the extent to which people experienced LFT dropped quickly (i.e., within a week after social distancing restrictions were lifted), but did not decrease further when all restrictions were lifted. In line with this, a significantly higher percentage of participants experienced higher levels of LFT during the pandemic, whereas the prevalence of lower levels of LFT was higher in the post-pandemic studies. However, there was no significant difference between the overall prevalence of LFT during the pandemic (86.1%) and post-pandemic (80% and 78.2%).

The findings regarding the severity of LFT are largely compatible with previous research. First, the extent to which people experience LFT has been associated with the presence of social distancing regulations^4^ and the duration of these regulations^3^. It could therefore be expected that removal of social distancing regulations would result in more opportunities for interpersonal touch, which consequentially would lead to a reduction in LFT. This is consistent with the significant difference between pandemic and post-pandemic LFT severity. Interestingly, severity was not lower in the second post-pandemic study but rather seemed to remain stable. It is therefore important to continue monitoring LFT severity in the general population to see if this stability remains over longer periods of time. However, it is important to note that other factors could explain the significant difference between the pandemic and post-pandemic data, aside from the absence of social distancing regulations. The samples could, for example, also have differed with respect to attitudes towards touch, the size of their social networks or living conditions. It was beyond the scope of the current paper to include additional covariates, and the limitations of this approach will be discussed more extensively later on.

These results are also generally consistent with observations regarding the prevalence of LFT. The prevalence of high levels of LFT was found to be significantly higher during the pandemic, which reflects the elevated LFT severity observed during the this time. The insignificant difference in the overall prevalence of LFT is somewhat surprising, but does indicate that a largely number of individuals continue to experience a desire for more interpersonal touch in the absence of social distancing regulations. These findings also emphasize the importance of looking at both prevalence and severity simultaneously: despite the high prevalence, LFT severity is still significantly lower in both post-pandemic samples compared to the levels that were observed during the pandemic. Including both parameters can therefore provide a more complete understanding of how LFT is experienced in the general population, and how this changes over time.

Although unexpected, these findings signal that, even outside of the pandemic, more than three quarters of people report that they are not experiencing social touch as often as they would prefer. This seems to be corroborated by the aforementioned findings of Beßler et al.^5^. Together, these results suggest that a large portion of the general population experiences LFT to a certain extent. A rich body of literature shows that our sense of touch does not only enable us to perceive the world around us, but is also crucial for forming social connections and strengthening bonds^13^. Touch is even thought to play an important role in our (mental) health: it has, for example, been shown that social touch has a positive impact on pain^14^ and stress relief^15^. Moreover, a lack of social touch has been associated with increased feelings of depression, worse general health^16^ and reduced psychological, physical and social wellbeing^2^. We therefore strongly advocate that it is important to continue monitoring LFT and the potential consequences in the general population in future research. In addition, we believe that it is vital to understand and identify the causes of these high levels of LFT. There is currently no systematic overview of potential barriers to touch, aside from data about COVID-19 related restrictions. However, it is likely that a wide variety of factors is involved. Examples of possible avenues for future research could be the influence of increased online communication in the digital age, and societal shifts in attitudes towards touch perception in the wake of the #MeToo movement^17^. Identifying potential barriers is crucial for developing more efficient ways to reduce LFT in the future.

There are, however, a few important limitations that need to be considered. As mentioned before, several individual factors may predict the development of LFT^18^. We only considered age and gender as possible covariates in the current study, but other variables are likely to be involved. As mentioned before, examples could be attitudes towards touch, the size of social networks or someone’s living conditions. Identifying predicting factors in a post-COVID-19 society is important to more efficiently find and help those at risk of developing LFT. Including additional covariates would also have provided more certainty about the nature of the observed differences between the COVID and post-COVID data. This was not possible due to a limited overlap between demographic questions and psychological measures. This means that, even though we resampled pandemic data, it remains possible that the observed results could be caused by differences in sample constitution. However, it should be noted that the primary aim of the current paper was to explore the severity and prevalence of post-pandemic LFT, rather than providing an in-depth analysis of factors that can explain fluctuations in LFT data. Second, data from different samples were compared, which impairs the ability to statistically compare data and thus the interpretation of the results. Nevertheless, in the absence of existing longitudinal research, the current study is an important starting point for future research. It emphasizes the need for these studies by drawing attention to the aforementioned and consistently high prevalence of LFT observed before, during and after the pandemic. Future studies should specifically focus on collecting longitudinal data in the general population, to ensure that potential changes in LFT severity and prevalence can be monitored more accurately.

Third, the generalizability of the results are limited due to the characteristics of the included samples. The post-COVID samples primarily consisted of women, which could potentially have biased the results. It is therefore also not possible to draw conclusions about gender-related differences on the basis of the current results. In addition, participants who had been diagnosed with any form of psychopathology were excluded from the analysis. Future research is therefore needed to assess whether the prevalence and severity of LFT differs in various clinical populations. Post-COVID data was collected at science festivals, meaning that the sample might not be an accurate reflection of the general population. Taken together, future studies should aim to include more diverse and balanced samples in order to improve the generalizability of the results.

Lastly, the items included in the LFTQ are broad and might be interpreted differently by different participants. Despite a high internal consistency, the broad formulation of the items in the LFTQ might impact the reliability of the questionnaire and the extent to which results can be compared across participants. In future studies, this needs to be controlled by using measures in which participants can indicate to what extent they experience LFT in different social relations and with respect to different forms of interpersonal touch^5^.

To conclude, the current results suggests that the extent to which LFT was experienced was significantly higher during the COVID-19 pandemic than in after the -pandemic. Although these differences could be caused by the implementation of social distancing measures, alternative explanations cannot be ruled out. It was beyond the scope of the current study to determine potential causal or explanatory factors. The main aim of our study was instead to observe whether or not LFT continued to be present in a post-pandemic society. Our data clearly shows that the majority of participants still experienced LFT after the pandemic, even though the severity seemed to reduce. This suggest that LFT continues to require attention and that it is not an issue that is exclusively related to the COVID-19 pandemic. Given the well-documented benefits of frequent interpersonal touch, high levels of LFT are likely to have negative consequences for several pillars of general wellbeing. Future research is therefore needed to further investigate the implications of high levels of LFT in the general population and to identify potential barriers to touch.

## Data Availability

The data presented in this study are available on request from the corresponding author. The data are not publicly available due to ethical reasons; sharing data in a publicly accessible repository was not included in the informed consent form.
